# Distribution and impact of yeast thermal tolerance permissive for mammalian infection

**DOI:** 10.1186/s12915-015-0127-3

**Published:** 2015-02-26

**Authors:** Vincent Robert, Gianluigi Cardinali, Arturo Casadevall

**Affiliations:** Centraalbureau voor Schimmelcultures CBS, 8 Uppsalalaan, 3584CT Utrecht, The Netherlands; Department of Pharmaceutical Sciences, University of Perugia, Perugia, Italy; Department of Microbiology and Immunology and the Division of Infectious Diseases of the Department of Medicine of the Albert Einstein College of Medicine, 1300 Morris Park Ave, Bronx, NY 10461 USA

**Keywords:** Disease, Fungi, Mammal, Taxonomy, Temperature

## Abstract

**Background:**

From the viewpoint of fungal virulence in mammals, thermal tolerance can be defined as the ability to grow in the 35°C to 40°C range, which is essential for inhabiting these hosts.

**Results:**

We used archival information in a fungal collection to analyze the relationship between thermal tolerance and genetic background for over 4,289 yeast strains belonging to 1,054 species. Fungal genetic relationships were inferred from hierarchical trees based on pairwise alignments using the rRNA internal transcribed spacer and large subunit rDNA (LSU) sequences. In addition, we searched for correlations between thermal tolerance and other archival information including antifungal susceptibility, carbon sources, and fermentative capacity. Thermal tolerance for growth at mammalian temperatures was not monophyletic, with thermally tolerant species being interspersed among families that include closely related species that are not thermal tolerant. Thermal tolerance and resistance to antifungal drugs were not correlated, suggesting that these two properties evolved independently. Nevertheless, the ability to grow at higher temperatures did correlate with origin from lower geographic latitudes, capacity for fermentation and assimilation of certain carbon sources.

**Conclusions:**

Thermal tolerance was significantly more common among ascomycetous than basidiomycetous yeasts, suggesting an explanation for the preponderance of ascomycetous yeasts among human pathogenic fungi. Analysis of strain maximum tolerable temperature as a function of collection time suggested that basidiomycetous yeasts are rapidly adapting to global warming. The analysis identified genera with a high prevalence of the thermal-tolerant species that could serve as sources of emerging pathogenic fungi.

**Electronic supplementary material:**

The online version of this article (doi:10.1186/s12915-015-0127-3) contains supplementary material, which is available to authorized users.

## Background

Thermal tolerance is the capacity to of an organism to survive and replicate at relatively high temperatures. The fungal kingdom includes the most thermotolerant eukaryotic organisms known, several of which can replicate at temperatures up to 61°C [1]. The upper temperature limit for eukaryotes appears to be 62°C, which has been suggested as the highest temperature at which cellular membranes can remain both thermostable and functional [2]. Although the definition of thermal tolerance is relative to the subject and environment under study, we are primarily interested in the temperature range that allows certain fungi to find the mammalian host as a permissive environment for growth. With regards fungal virulence, the capacity of fungal species to cause disease is a function of their ability to survive at the host temperatures. This phenomenon is vividly illustrated by the emergence of a new disease in bats known as white nose syndrome that is caused by a fungus that thrives in cooler temperatures, *Pseudogymnoascus destructans* [3]. In summer months the higher body temperatures of bats excludes the fungus but during the winter months the bats hibernate and their temperature drops, making them vulnerable to *P. destructans*. White nose syndrome is lethal unless the bats are wakened from the torpor state and placed in a supportive environment, where higher temperatures restrict the growth of the fungus, curing the bats [4]. Similarly, the chytrid fungus *Batrachochytrium dendrobatidis* is decimating amphibian populations worldwide, but those species with higher temperatures are less vulnerable [5] and diseased frogs can be cured by placing them in a supportive environment at 37°C [6,7].

Because we are primarily interested in mammalian fungal diseases, we will use the temperature range of advanced mammals (35°C to 40°C) as our working definition of fungal thermal tolerance. A prior analysis of fungal thermal tolerances revealed that the majority of fungal species grew at temperatures <30°C, but there was a rapid decline in the number of fungal species that were able to grow in the 30°C to 40°C range [8]. This suggested that one explanation for the relative paucity of fungal diseases in mammals could be resistance associated with mammalian endothermy and homeothermy coupled with vertebrate adaptive immunity. Evidence for the synergistic combination of temperature and immunity come from the rabbit model for cryptococcosis, where their high basal body temperature makes them invulnerable to systemic disease unless immunosuppressed, although local infection is possible in cooler body regions [9,10]. Similarly, host temperature is a major variable determining the susceptibility of moths to cryptococcal species [11]. When fungal thermal susceptibility data was analyzed in the context of the energy cost of mammalian endothermy, the results suggested that mammalian temperatures corresponded to an optimum where the majority of fungal species were thermally excluded relative to the caloric costs required to maintain such elevated temperatures [12]. This information, together with the relative resistance of mammals to fungal diseases, led to the proposal that mammals emerged from the cataclysm that marked the end of the Cretaceous as a result of fungal selection, which favored endothermic mammals relative to ectothermic reptiles [13,14].

Fungi are currently devastating major ecosystems and represent a threatening source of potential pathogens for humans [15]. Given that fungal thermal tolerance at mammalian temperatures is an absolute requirement for virulence to mammals, there is considerable interest in mapping the thermal tolerances of fungi to identify those with potential for pathogenicity. This topic is of increasingly urgent importance because climate warming could lead to the emergence of new fungal diseases as fungi adapt to survive in a warmer environment by becoming more thermotolerant [16]. Evidence for the ability of fungi to rapidly adapt to higher temperature includes an observation that an insect pathogenic fungus could be adapted to survive at much higher temperatures in an effort to improve its pest-control potential by defeating the capacity of insects to protect against this pathogen by behavioral fevers [17]. In recent years we have seen several outbreaks of fungal diseases in humans, including the continuing epidemic of *Cryptococcus gattii* in North America [18], fungal keratitis in contact lens users [19], and steroid injection-associated *Exserohilum rostratum* fungal meningitis [20], highlighting a continued threat from new pathogens from the fungal kingdom.

Despite substantial work on the area, the molecular and cellular mechanisms responsible for fungal cell survival at higher temperatures remain enigmatic because of their complexity [21]. In this study we correlated thermal tolerance with genetic information to reveal the taxonomic relationship between fungal species with regards their ability to grow at different temperatures. This type of analysis can provide clues to the evolution of thermal tolerance and identify those fungal taxa with the potential to generate new mammalian pathogenic fungi. Historically, this type of analysis has provided insights into the evolution of virulence in fungi. For example, a phylogenetic analysis of human pathogenic fungi and their close relatives led to the suggestion that the capacity for virulence probably arose independently several times in the course of fungal evolution [22]. Furthermore, understanding of the evolutionary interactions between thermotolerance, energetic catabolism and drug resistance can give useful insights into the relationships between these mechanisms required for the successful growth of these organisms in the challenging environment defined by animal bodies reacting to infections.

## Results

### Taxonomic distribution of thermal tolerance

**Figure 1:**
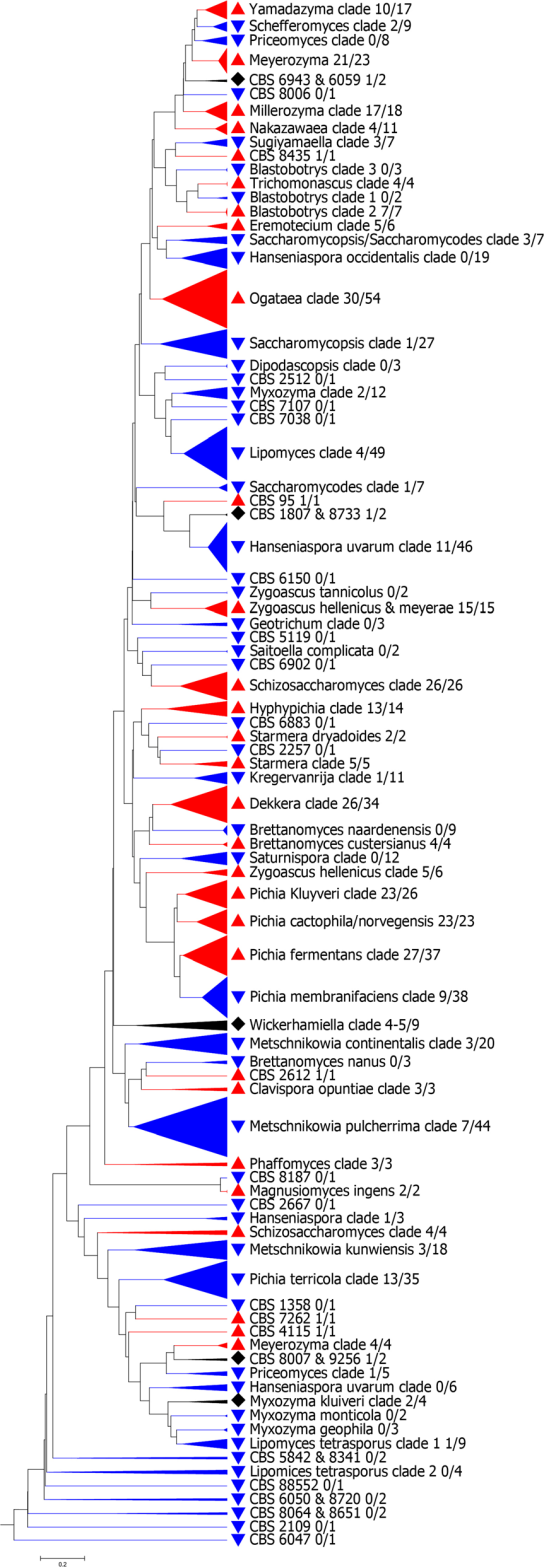
**Unweighted Pair Group Method with Arithmetic Mean tree of ascomycetous yeasts not belonging to the Saccharomycetaceae family obtained from a distance matrix based on pairwise alignments of the ITS (ITS1-5.8S-ITS2) and 26S (D1-D2) loci.** The scale bar represents the distance (0.2 means 20% distance) between the nodes of the tree.

**Figure 2:**
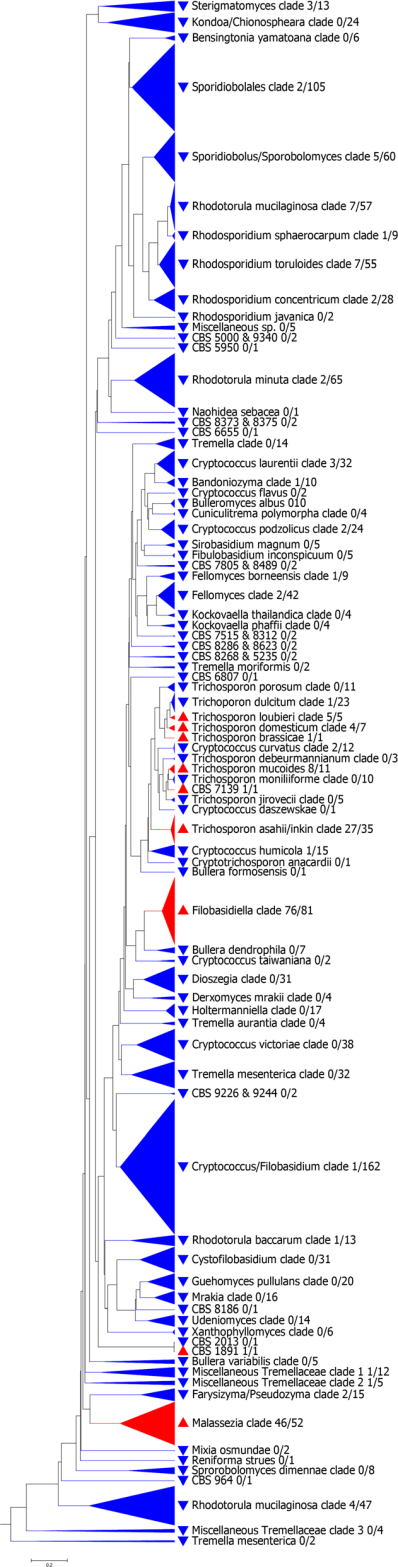
**Unweighted Pair Group Method with Arithmetic Mean tree of basidiomycetous yeasts obtained from a distance matrix based on pairwise alignments of the ITS (ITS1-5.8S-ITS2) and 26S (D1-D2) loci.** The scale bar represents the distance (0.2 means 20% distance) between the nodes of the tree.

Analysis of hierarchical trees generated from the rRNA internal transcribed spacer (ITS) and 26S sequence data as a function of the ability of strains to grow at higher temperatures revealed that thermal tolerance is not monophyletic (Figures 1 and 2, Additional file 1: Figure S1 and Additional file 2: Figure S2). We are aware that the ITS locus cannot be used for large-scale phylogenetic tree reconstruction involving very diverse groups of individuals. We therefore opted instead for a phenetic-based approach using pairwise sequence alignments and selected the Unweighted Pair Group Method with Arithmetic Mean (UPGMA) [23] as a common and well-established clustering method. We made a few phenetic trees using other clustering methods, such as neighbor joining, and the results were essentially identical to the ones obtained with UPGMA; thermal-tolerant species were interspersed among non-thermal tolerant species (data not shown). Hence, the trees shown should be viewed in light of these limitations and not used to infer phylogenetic relationships. Instead, they can be viewed as indicating that thermal tolerance was neither monophyletic nor homogenously distributed among fungal genera. Some genera were consistently able to grow at higher temperatures. Genera for which the number of strains available was equal or higher than 10 with more than 50% of strains growing at 37°C or higher are listed in Table 1, and the major sources of such strains are listed in Table 2. Thermal-tolerant strains were significantly more common (*P* < 0.0001, Chi-Square) among the ascomycetes (1,257 out of 2,868, 44%) than for the basidiomycetes (215 out of 1,421, 15%). Furthermore, analysis of the maximum growth temperature and phylum revealed a significant association (Table 3).

**Table 1:** **Thermotolerant fungal phyla and genera**

**Phylum**	**Genera**	**Number strains/total**	**Percentage**	**Species of medical interest** ^**a**^
Ascomyces	All species	1,257/2,868	44%	ND
	*Blastobotrys*	64/80	80%	2
	*Clavispora*, Metschnikowiaceae	11/11	100%	1
	*Cyberlindnera*	47/75	63%	1
	*Dekkera*	23/32	72%	0
	*Kodamaea*	21/22	95%	1
	*Kluyveromyces*	75/98	76%	2
	*Lodderomyces*	9/10	90%	0
	*Millerozyma*	28/28	100%	1
	*Meyerozyma*	26/28	93%	2
	*Pichia*	112/202	55%	3
	*Phaffomyces* and *Starmera*	11/12	92%	0
	*Saccharomyces*	196/306	64%	1
	*Schizosaccharomyces*	26/27	96%	0
	*Zygoascus*	17/17	100%	0
Basidiomycetes	All species	215/1,421	15%	ND
	*Filobasidiella*	75/77	97%	7
	*Malassezia*	45/54	83%	14

^a^Species of medical interest inferred from the following sources [24]. ND, not determined.

**Table 2:** **Major sources of thermal-tolerant strains**

**Source**	**Thermal tolerant/Total**	**Percentage**
Alpechín^a^	6/6	100%
Animals (non-human, non-bird)	69/101	68%
Birds	18/30	60%
Distilleries	19/22	86%
Effluents	7/11	64%
Fodder	6/12	50%
Human	269/413	65%
Milk	5/9	56%
Wine	64/105	61%

^a^Waste from production of olive oil.

**Table 3:** **Coefficients of correlation between thermal tolerance and other selected parameters**

**Parameter A**	**Parameter B**	**Correlation coefficient**		
		**Kendall**	**Pearson**	**Spearman**
Maximum growth temperature	Phylum (asco-, basidiomycetes)	0.405	0.469	0.460
Latitude (absolute value)	Maximum growth temperature	-0.080	-0.144	-0.110
Latitude (absolute value)	Growth at 4°C	0.178	0.202	0.217
Latitude (absolute value)	Growth at 18°C	0.070	0.094	0.084
Latitude (absolute value)	Growth at 25°C	-0.147	-0.216	-0.178
Latitude (absolute value)	Growth at 30°C	-0.449	-0.500	-0.551
Latitude (absolute value)	Growth at 35°C	-0.210	-0.208	-0.265
Latitude (absolute value)	Growth at 37°C	-0.217	-0.217	-0.265
Latitude (absolute value)	Growth at 40°C	-0.078	-0.057	-0.093
Caspofungin resistance	Growth at 37°C	-0.279	-0.322	-0.316
Fluconazole resistance	Growth at 37°C	-0.185	-0.219	-0.209
Glucose fermentation	Growth at 37°C	0.313	0.327	0.327
Growth at acidic pH	Growth at 37°C	0.291	0.297	0.301
Urea hydrolysis	Growth at 37°C	-0.273	-0.273	-0.274
Diazonium Blue B reaction	Growth at 37°C	-0.302	-0.303	-0.304
Glucose assimilation	Growth at 37°C	-0.010	-0.017	-0.010
Galatose assimilation	Growth at 37°C	0.069	0.062	0.071
Maltose assimilation	Growth at 37°C	0.014	0.012	0.015
Sucrose assimilation	Growth at 37°C	0.012	0.008	0.012
Trehalose assimilation	Growth at 37°C	-0.050	-0.052	-0.052
Melibiose assimilation	Growth at 37°C	-0.128	-0.124	-0.131
Lactose assimilation	Growth at 37°C	-0.139	-0.132	-0.143
Palatinose assimilation	Growth at 37°C	-0.023	-0.002	-0.024

The genus *Candida* is responsible for most human fungal infections, but this classification is increasingly meaningless. Molecular evidence reveals that *Candida* should be split into many genera, because it is definitely not monophyletic according to any taxonomic marker. With that caveat in mind, the genus *Candida* was not highly thermotolerant as a whole, with only 342 of 810 (42%) strains being able to grow at 37°C or above. However, some clades of this genus, identified by rRNA sequence data, manifested considerable thermal tolerance. These included *Candida albicans* (65/65 strains, 100%), *Candida catenulata* (8/12, 67%), *Candida dubliniensis* (4/4, 100%), *Candida glabrata* (11/11, 100%), *Candida haemulonii* (7/7, 100%), *Candida magnoliae* (8/11, 73%), *Candida odintsova* (7/9, 78%), *Candida parapsilosis* (14/14, 100%), *Candida pararugosa* (5/5, 100%), *Candida rugosa* (6/6, 100%) and *Candida tropicalis* (23/25, 92%).

Other ascomycetous species like *Millerozyma farinosa* (27/27, 100%), *Dekkera anomala* (6/11, 55%), *D. bruxellensis* (17/21, 81%), *Pichia cactophila* (11/11, 100%), *P. fermentans* (7/14, 50%), *P. holstii* (8/12, 67%), *P. kluyveri* (12/13, 92%), *P. norvegensis* (15/15, 100%), *Kluyveromyces lactis* var. *drosophilarum* (16/16, 100%), *K. marxianus* (41/41, 100%), *Lachancea fermentati* (12/12, 100%), *Saccharomyces cerevisiae* (193/210, 95%), *Torulaspora globosa* (10/10, 100%), *Schizosaccharomyces pombe* (12/12, 100%), *Cyberlindnera jadinii* (12/12, 100%), *Kodamaea ohmeri* (14/14, 100%), *Lodderomyces elongisporus* (9/10, 90%), *Zygoascus hellenicus* (9/9, 100%) and *Z. meyerae* (8/8, 100%), were also consistently thermotolerant.

Among the basidiomycetous yeasts, only a few species like *Cryptococcus bacillisporus* (75/77, 97%), *C. neoformans* (46/46, 100%), *Trichosporon asahii* (13/13, 100%), *T. inkin* (8/8, 100%), *Malassezia furfur* (16/16, 100%), *M. globosa* (6/9, 67%), *M. pachydermatis* (11/11, 100%), *M. slooffiae* (4/5, 90%) and *M. sympodialis* (6/6, 100%) are outstanding in terms of their ability to grow at high temperatures.

### Thermal tolerance and geographic origin

Because the Centraalbureau voor Schimmelcultures (CBS) database included information on the geographic origin of the strains, we analyzed our dataset for correlations between the geographic origins of the strains and their ability to grow at higher temperatures. Localities listed as strain source origin were automatically transformed into latitude and longitude (degree radians) using the GPS Visualizer web service [25]. For the latitude, their absolute degree radians values were correlated with their ability to grow at various temperatures. We found a positive correlation between the ability to grow at higher temperature and isolation localities with small absolute latitudes. The correlations between the maximum growth temperatures and the absolute latitude were weak (Table 3). However, a different picture emerged when we accounted for the ability to grow at different temperatures. For example, a much stronger correlation emerged when we analyzed the ability to grow at 30°C and the absolute latitude (Table 3). For temperatures below and above 30°C, the magnitude of the correlation values tended to decrease gradually (Table 3). Although our data do not allow us to draw definitive conclusions between thermal tolerance and latitude of isolation, we did find that tropical strains were more likely to sustain higher growth temperatures than strains found at higher absolute latitudes. In addition, more basidiomycetous yeasts originated from locations at higher absolute latitudes than ascomycetous yeasts (0.341, 0.412, and 0.343 for the Pearson, Spearman and Kendall correlations, respectively).

### Growth at temperature extremes

**Figure 3:**
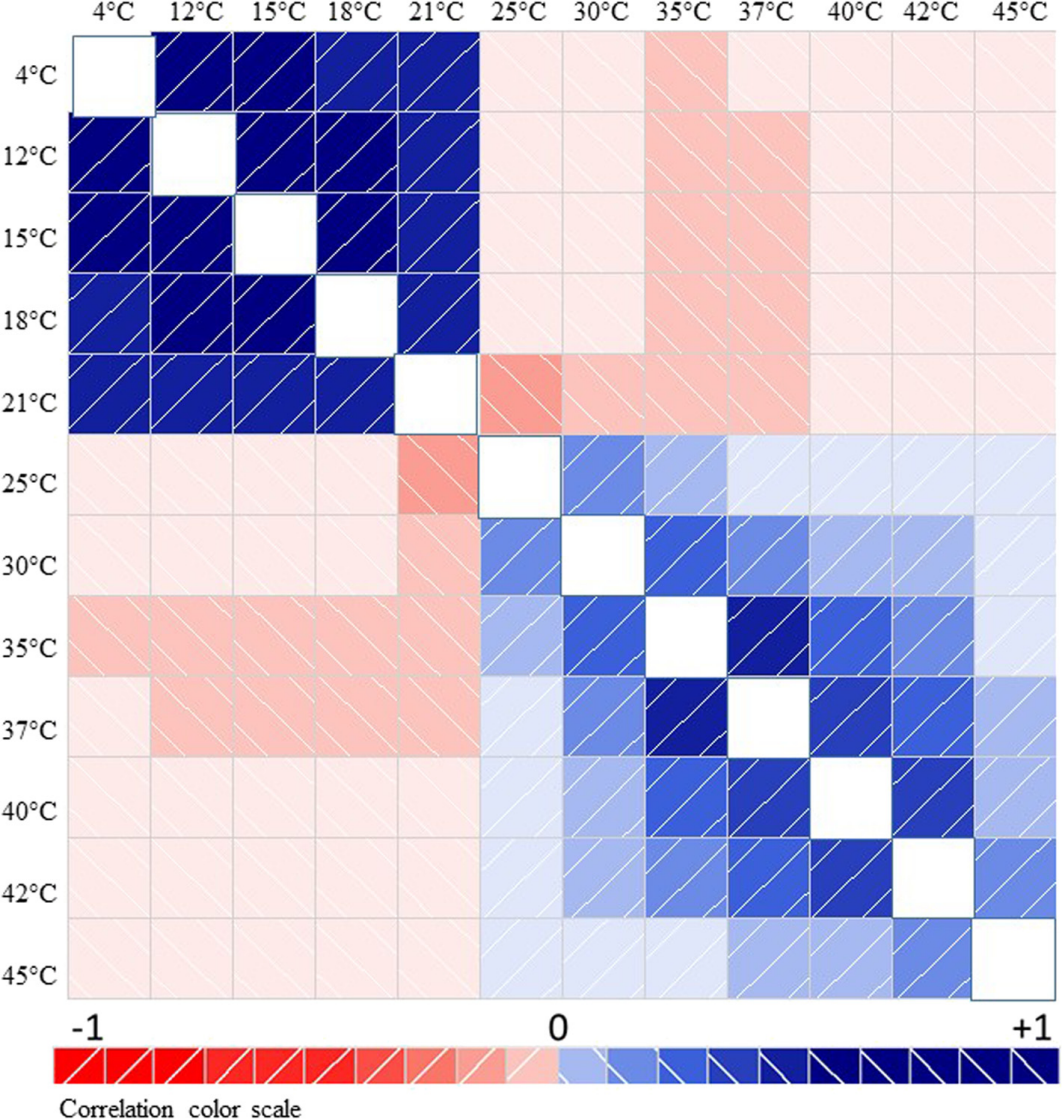
**Diagram depicting the correlation of growth behavior at different temperatures by the fungal species studied.** Blue and red colors denote areas of positive and negative correlation, respectively. Correlation absolute values are indicated by color intensity (bottom panel). Please see Additional file 4: Figures S4 for additional details on the correlation between color and statistical significance.

We also used the database to evaluate several hypotheses regarding tolerances at thermal extremes. We hypothesized that the physiological mechanisms regulating optimal growth at lower temperatures and resistance to cold were different from those regulating optimal growth at higher temperatures and resistance to heat. This hypothesis was tested by correlating the growth behavior of all strains at the various temperatures and by reporting the correlation values according to a color scale in a correlogram for easier reading (Figure 3; Additional file 3: Figure S3). The correlogram shows two blocks of positive (blue) correlations: one including temperatures between 4°C and 21°C and the other from 25°C to 45°C. The correlogram reveals the absence of positive and/or significant correlations between growth at lower temperatures (<21°C) and at higher temperatures (>25°C). The absence of such a correlation indicates that the resistance to, and the growth intensity at, different temperatures is governed by two distinct processes, one prevailing in basidiomycetes (<21°C) and the other in ascomycetes (>25°C). The discontinuities between the temperature ranges suggest two general and potentially different mechanisms for coping with temperature stress that may be evolutionarily divergent. Interestingly, the block at lower temperatures is characterized by positive and strong correlations (>0.78), whereas the block at higher temperatures has a correlation of 0.01 between 25°C and 45°C and a correlation of 0.72 between the growth at 35°C and 37°C. This distribution of correlation values indicates a much higher variability in the growth behavior at higher temperatures than that observed below 21°C, suggesting that mechanisms responsible for fungal tolerance at higher temperatures are more diverse than those operating at lower temperatures.

### Thermal tolerance and biochemical characteristics

The CBS database also contained information on biochemical characteristics of the strain set analyzed, so we searched for correlations between thermal tolerance and various biochemical tests. Positive correlations were observed between maximum growth temperature and the abilities to ferment D-glucose and to grow at the acidic pH of 3.0 (Table 3). By contrast, negative correlations were observed for urea hydrolysis and the Diazonium Blue B reaction (Table 3). The Diazonium Blue B reaction differentiates between ascomycetous and basidiomycetous yeasts [26]. The negative correlation for ureases activity is consistent with the fact that these strains were mostly basidiomycetous yeasts that have lower overall thermal tolerances.

**Figure 4:**
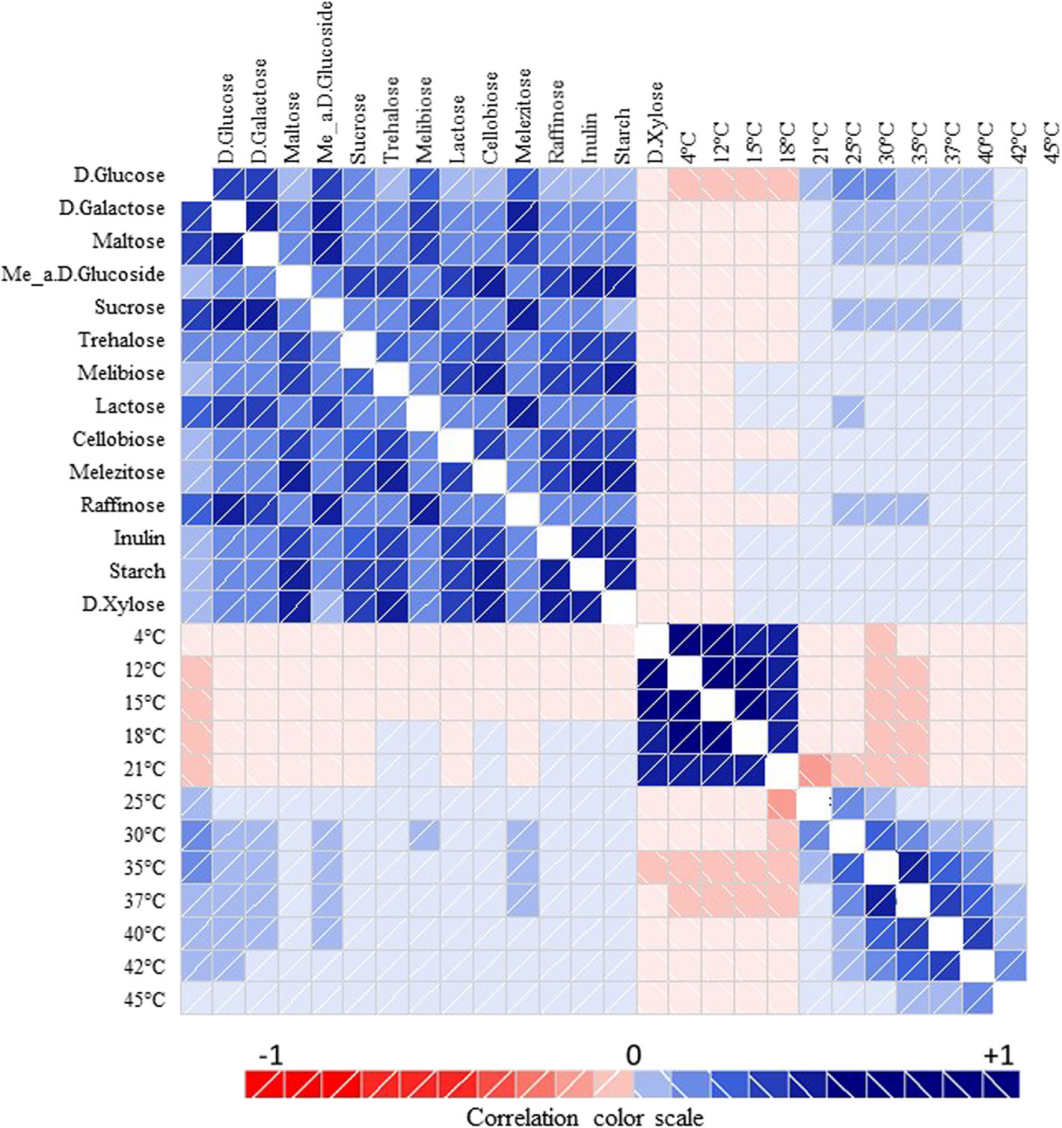
**Diagram depicting the correlation between the growth intensity of the fungal species studied on fermentable C sources and on glucose at different temperatures.** Blue and red colors show positive and negative correlation, respectively. Correlation absolute values are indicated by color intensity.

We hypothesized that aerobic respiration was more likely to be found and thus correlated with organisms that grew well at lower temperatures, whereas fermentation was present in fungal strains able to tolerate higher temperatures. A rationale for this hypothesis is the fact that fermentation in *S. cerevisiae* has a much higher sugar consumption rate (up to 5×) than the respiration per unit of produced biomass, and thus should produce more heat per unit of time [27]. Fungal fermentation is more common with sweet substrates and in warm environments. This hypothesis was further tested with a correlogram, which noted that fermentation was positively correlated with the ability to grow at higher temperatures on glucose and negatively with lower temperatures (Figure 4). The ability to ferment glucose correlated negatively with temperatures up to 18°C and positively with those over 21°C, confirming that for the fungal growth on glucose a discontinuity exists between 21°C and 25°C. For most of the other sugars, the border between negative and positive (although weak, from -0.2 to 0.2) correlation was between 18°C and 21°C as for glucose and between 12°C and 15°C for melibiose, lactose, melezitose, inulin, starch and D-xylose. Interestingly, two of these sugars, lactose and melibiose, are anomers of glucose and galactose, which are hydrolyzed and transported with different mechanisms in fungi, with both requiring the Leloir Pathway to introduce galactose into glycolysis and sometimes some additional futile cycles [28]. Inulin and starch are long-chain polymers demanding strong enzymatic hydrolysis prior to fermentation. These observations suggest that the shift to the lower temperatures observed for these sugars could be justified by complex mechanisms acting before hydrolysis, and therefore slowing overall metabolism. Metabolism of certain organic acids, such as isomaltulose,was highly positively correlated with the ability to grow at lower temperatures, and negatively correlated with the ability to grow at higher temperatures, while other carbon sources showed differences in their correlation with growth in different temperature ranges (Table 3).

### Thermal tolerance and antifungal susceptibility

Since both heat and antifungal agents produce forms of stress on fungal cells, we investigated whether there was a correlation between thermal tolerance and antifungal drug susceptibility. We found a negative correlation between caspofungin resistance and maximum growth temperature (Table 3; Additional file 4: Figure S4). Caspofungin resistance was quite strongly correlated with thermal tolerance for the basidiomycetes (0.758 for Pearson, Spearman and Kendall). Similarly, a negative correlation with maximum growth temperature was found for fluconazole but it was weaker than that observed for caspofungin (Table 3). Other azoles drugs showed a similar pattern but with weaker correlations.

### Thermal tolerance trends over time

The data on the maximum and minimum temperature of growth of the CBS strain collection cover close to a century, with the oldest dating back to 1915. Although the strains were not evenly collected over the years and some periods (for example, during World War Two) are almost without records, the data available were sufficient to pose the question on whether the global temperature affects the maximum temperature of growth (hereinafter referred to as T_max_) of the collected strains. Here, it must be stressed that temperatures of growth were analyzed and recorded shortly after strain isolation, thus ensuring that the conditions of the subsequent handling by the culture collection did not affect these data. To decrease the inevitable random variations caused by the amounts of strains introduced in the CBS collection in the various years, 10-year mobile averages were calculated, yielding smoother and more readable curves. The global temperature mean, obtained from the National Aeronautics and Space Administration website [29] and reported as temperature index with a base period 1951 to 1980, shows a well-known increase after 1980, consistent with the concept that a significant trend of global warming started during that time period [30]. During the last century, the ascomycetous T_max_ ranged from about 32°C to 35°C with a maximum in the period between 1960 and 1970. The variations observed for the basidiomycetous yeasts were almost twice those of the ascomycetes, ranging between 26°C and 32°C. The decrease in the basidiomycetes T_max_ observed between 1970 and 1980 can be partly ascribed to the very low number of strains introduced in the CBS collection during that time span. In general, the most visible trend between 1920 and 1980 is an increase in the difference in T_max_ between ascomycetes and basidiomycetes. The very nature of the data available, and the fact that they were not collected originally for the purpose of the present analysis, does not allow us to draw conclusions on this trend, surely affected by a general reduction of isolation in the period between 1960 and 1980. During the last 30 years, a rapid increase of the basidiomycetes T_max_ could be observed, whereas the ascomycetes T_max_ remained around 33°C on average.

**Figure 5:**
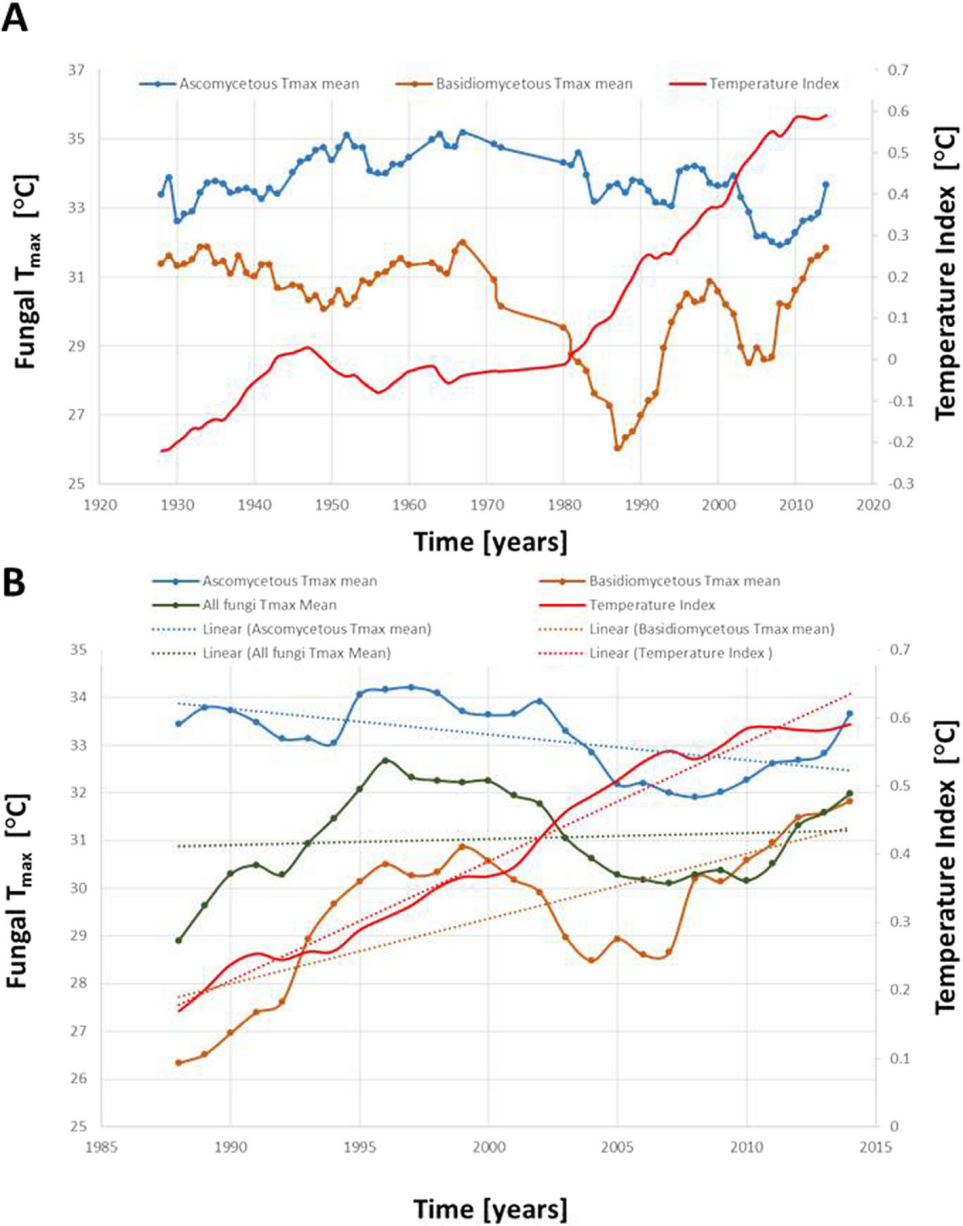
**Trends in strain Tmax over time. (A)** Trend of fungal maximum temperature of growth T_max_ during the last century, compared with the global temperature index. The fungal T_max_ reported was calculated from a 10-year mobile mean. The temperature index is the global mean land-ocean temperature index, 1880 to present, with the base period 1951 to 1980. **(B)** Regression analysis of fungal T_max_ and the global temperature index during the last 30 years.

To test whether global warming could have affected T_max_ of the basidiomycetes, but not ascomycetes, a linear regression analysis was carried out using the available T_max_ and global temperature index data series in the 26 years spanning 1988 to 2014 (Additional file 2: Figure S2). Ascomycetes showed a shallow decrease with very little statistical support (r^2^ = 0.34), corresponding to -0.0088°C per year (equivalent to 0.88°C per century), indicating a wavy yearly trend without substantial changes. By contrast, basidiomycetes T_max_ increased by 5.5°C, corresponding to 0.211°C per year (21.11°C per century). This was supported by a steeply increasing regression curve with r^2^ = 0.50. When ascomycetous and basidiomycetous were analyzed together, the T_max_ increase was 0.11°C per year, depicted by an increasing although not steep, regression curve with negligible r^2^ (0.01). Restricting these analyses to the period between 1995 and 2014 (mobile averages ranging from 2005 to 2014), when a much more consistent amount of strains and data was available, ascomycetes and basidiomycetes showed 1.49°C and 2.90°C T_max_ per year increases, respectively (Figure 5). These trends were supported by the linear regressions, with r_2_ = 0.66 for ascomycetes and 0.92 for the basidiomycetes. Altogether, these data strengthened the hypothesis that global warming could have caused an increase in basidiomycetes T_max_, with an only slightly significant effect on ascomycetes.

## Discussion

We took advantage of bioinformatics tools and the information archived at the CBS collection to investigate the distribution of thermal tolerance among yeast species. This analysis is a follow-up of a prior study where we showed that the majority of fungal species could not grow at mammalian temperatures, a finding interpreted as supporting the notion that vertebrate endothermy is a major defense mechanism against fungal diseases [8]. Because we are primarily interested in identifying fungal genera that could serve as sources of new human fungal pathogens, we used the ability to grow at 35°C to 40°C as our operational definition of thermal tolerance. Hierarchical trees were obtained using rDNA sequence data, thus avoiding ambiguities, uncertainties and inaccuracies associated with genus and species names, especially in some genera like *Candida*. Furthermore, we took advantage of the fact that many of these isolates had been tested for antifungal drug susceptibility [31] to explore whether there was any correlation between thermal tolerance and drug resistance. The CBS database also contains a large number of additional physiological data associated with the strains and we have tried to correlate maximum growth temperatures to other strains characteristics such as fermentation, sugar utilization and carbon source assimilation. By combining these data we have produced the first analysis of the thermal tolerance across yeast species. The results provide important insights into the evolution of this trait and potential sources of new fungal pathogens.

Thermal tolerance among fungal species was not monophyletic. Thermal-tolerant fungal species are found interspersed with non-thermal tolerant species in all families. Thermal-tolerant species are closely related to species that manifest no thermal tolerance, and a single thermal-tolerant species was sometimes found in the midst of many related species that are not thermal tolerant. This suggests that either the capacity for thermal tolerance has arisen independently several times in evolution, that thermal-tolerant species represent a set of fungi that retains an ancestral trait selected for in a distant past when the Earth was much warmer, or both. We cannot discriminate between these possibilities with the data at hand. For much of the Earth’s history, the climate was much warmer than in recent geologic epochs, when the planet cooled by about 5°C in the past 34 million years [32]. Hence, thermal tolerance may be a retained trait from a much warmer time. On the other hand, experiments with directed evolution of a fungal species have shown that it is relatively easy to breed a strain for growth at higher temperatures [17], suggesting that some fungi are highly adaptable, and hotter microclimates, such as those that occur in composting, fermentation and/or thermally extreme environments could have selected for thermal tolerance. The similarity between *S. cerevisiae* strains recovered from human vagina and those found in fermentative industrial environments are consistent with the notion that environmental adaption to higher temperatures can allow species to acquire the capacity for mammalian infection [33].

In recent years there has been an explosion in fungal species associated with disease in vertebrate animals [15,34]. Many of these new fungal pathogens have been associated with disease in individuals with impaired immunity, whereas the agents of white nose syndrome in bats and chytridiomycosis in frogs cause disease in otherwise healthy populations. Fungal diseases are relatively common in insects and ectothermic vertebrates but relatively rare among mammals, which are endothermic and homoeothermic. The paucity of fungal diseases in mammals may be the result of the combination of higher temperatures and adoptive immunity. Our analysis shows that genera that already include fungal pathogens also contain close relatives that are not thermal tolerant. Close relatives of pathogenic fungi that are currently not pathogenic could acquire pathogenic potential as a result of increased thermal tolerance through adaptation to global warming and/or deliberate directed evolution. However, genera with thermal-tolerant species that do not currently include mammalian pathogenic species should not be ignored because the capacity of mammalian virulence could emerge by selection from environmental forces, such as non-vertebrate hosts like amoeba and/or recombination events [35,36]. Hence, both genera that already contain pathogenic species and genera with many thermal-tolerant species warrant attention as new potential sources of human pathogenic fungi.

The fact that many thermally tolerant species are not yet associated with mammalian diseases implies that thermal tolerance is a necessary but not sufficient condition for fungal virulence to mammals. In this regard, it is noteworthy that microbial virulence requires a susceptible host. The emergence of some species as pathogenic fungus can reflect specific changes in host populations that increase vulnerability to infection and disease, such as the immunosuppression needed for successful organ transplantation [37]. We observed major differences in the prevalence of thermal-tolerant strains among the basidiomycetous and ascomycetous yeasts. The majority of the basidiomycetous yeasts (85%) were not able to grow at high temperatures whereas almost half of the ascomycetous yeasts (44%) grew at higher temperatures. This difference could explain why the majority of human fungal pathogens are ascomycetes. Ascomycetes families with non-thermal-tolerant fungi capable of growth at near-mammalian temperatures could be sources of new mammalian-pathogenic fungi if continued global warming results in selection of more thermal-tolerant species. In this regard, the finding that more basidiomycetous yeast originated from higher latitudes is consistent with the overall lower thermal tolerance of this phylum. Furthermore, the fact that most pathogenic fungi are associated with warmer latitudes [16], and the finding that tropical strains are more likely to tolerate higher growth temperatures than strains found at higher latitudes, is consistent and supportive of the notion of adaptability and/or selection by environmental temperatures. The difference in the prevalence of thermal-tolerant strains between ascomycetous and basidiomycetous yeasts was surprising and unexpected. In the absence of our data, one might have hypothesized that because both phyla occupy similar niches in the environment, they are presumably subjected to similar thermal selection pressures, and that there would be no major differences between these phyla. Consequently, the existence of such thermal-tolerance differences suggests the possibility that differences in the micro-environments occupied by members of each phyla result in exposure to different thermal selection pressures. There is a suggestion in our data that this may be the case, because more basidiomycetous yeasts originated from geographic locations with higher latitudes than ascomycetous yeasts. Differences in the global distribution of ascomycetous and basidiomycetous yeasts could explain differences in thermal tolerance based on ambient temperature selection but this conclusion must await dedicated sampling studies for fungal populations as a function of latitude. The mechanisms responsible for the differences in thermal tolerance between these phyla are not understood. Although higher GC content is associated with thermal extremophiles, this mechanism is not likely to be operative at ambient temperatures. In fact, basidiomycetous yeasts have higher GC content than ascomycetous yeasts [38].

We attempted to gain some insight into whether the mechanisms involved in growth at the lower and higher temperature ranges were the same by analyzing the strength of the correlations as a function of temperature for the strain set studied here. We found that the ability to grow and survive at a given temperature was highly correlated in regions between 4°C and 21°C and between 30°C and 45°C. These regions were separated by an intervening region where correlations were weaker. Basidiomycetous yeasts were more frequent in the lower temperature range, whereas ascomycetous yeasts predominated in the higher temperature range. Although it is difficult to make definitive conclusions from thermal-tolerance correlation data, the discontinuity between low and high thermal tolerances suggests the possibility that the mechanisms underlying thermal tolerance in the two regions are different and evolutionarily divergent. This possible evolutionary divergence between high and low thermal tolerances was relatively strongly linked with the ability to ferment sugars at higher temperatures, suggesting that the traits necessary to grow at higher temperatures and to ferment sugars co-evolved. We have previously noted that fermentation produces more heat per time than assimilation, causing temperature increase in the absence of heat dissipation. This mechanism can be applied generally to all fungi, but another one can be peculiar of pathogenic organisms. In fact, when a fungal cell infects an animal body, the latter is simultaneously subject to higher temperature and to higher concentrations of CO_2_. The relative hypoxia and hypercarbia induce a decrease in respiration via the Pasteur effect, leading to fermentation that would not be otherwise possible owing to the low content of free glucose in the animal body [39-41]. Once fermentation starts, some species, such as *C. albicans*, manifest additional changes, such as a transition to hyphal growth, known to be triggered by low O_2_ and high CO_2_ content [42]. Although not all fungi of medical interest show the same features as *C. albicans*, our data suggest that the conditions favoring sugar fermentation and resistance to high temperature might have acted in concert during fungal evolution.

We investigated whether there was any correlation between susceptibility to antifungal drugs and thermal tolerance. The impetus for investigating this association was the assumption that both temperature and drug action have in common the fact that they produce stress in the fungal cell. We found a weak negative correlation between minimum inhibitory concentration of caspofungin and thermal tolerance. However, when basidiomycetous yeasts were considered separately, a strong correlation was observed. The molecular target of caspofungin is glucan synthase, an enzyme involved in synthesizing 1,3-β-glucan chains, which provide structural integrity to the cell wall. In *S. cerevisiae* there are two genes, *FKS1* and *FKS2*, which encode for alternative subunits of the 1,3-β-glucan synthase. These are known to be temperature regulated, with increased expression of FKS2 at higher temperatures [43]. Hence, the negative correlation observed between minimum inhibitory concentration and fungal tolerance could reflect differences in enzymatic activity at higher temperatures. Alternatively, these effects could be secondary to basidiomycetes cell wall since these organisms, as exemplified by *C. neoformans*, are often resistant despite having an echinocandin-susceptible enzyme [44,45]. In contrast to the weak association between antifungal drug susceptibility and thermal tolerance, we found strong correlations between thermal tolerance and fermentative capacity. Given that fermentation is a process associated with heat production, we interpret this association as suggesting a selection for ability to grow in higher temperature environment. Consistent with this notion, a high proportion of fungal strains isolated from wine and distilleries manifested thermal tolerance. However, it must be noted that there are some well-known exceptions to this generalization, including *C. neoformans,* which is a non-fermentative urease-producing basidiomycete [46].

The analysis of the available data on the T_max_ registered at the CBS collection during the last 99 years supported the idea that global warming can produce long-term increase in the maximum growth temperature, at least for the basidiomycetes. The fantastic increase of 21°C per century derived from the analysis of the last 26 years is definitely excessive and almost certainly reflects the relatively low T_max_ registered between 1960 and 1985, which in turn is likely due to random factors or to a paucity of basidiomycetes strains introduced into the CBS collection during that period. Nevertheless, these data were confirmed by analyzing a shorter period of 10 years when more data were available. Furthermore, the analysis of the period 1995 to 2014 showed that ascomycetes were also affected by global warming, although at a lower rate than the basidiomycetes. These findings suggest that global warming affects the basidiomycetes more, although a similar effect was observed more recently among the ascomycetes, which were likely to suffer less from global warming because they are in general more thermally tolerant. The T_max_ yearly rate of increase observed was much higher than the global temperature index for the same time periods. This fact can be likely explained by a massive and efficient selection of thermoresistant strains operated on by environmental factors. Whether this phenomenon is transient or lasting is not yet clear and cannot be predicted with the available dataset. However, the high value of the regression curves r^2^ for the data of the last 20 years is a clear warning that global warming may cause fungi to become significantly more thermally resistant, an event that could bring new fungal diseases [16], although predicting exactly which species will become threats may not be possible at this stage [11]. The importance of this topic at the general and medical level calls for an attentive and continuous monitoring of T_max_ data over time.

Analysis of archival data from thousands of isolates in culture collections using bioinformatics tools has the potential to provide insights that emerge only by comparing large samples. In fact, the insights obtained in this analysis would not be possible by analyzing smaller datasets, and it would be very difficult to assemble, organize and fund studies solely dedicated to obtaining fungal growth data as a function of temperature. However, this type of information is routinely obtained and archived when samples are deposited in collections and the dataset used here was gathered over many years. However, there are some limitations to this type of analysis that must be heeded in interpreting the findings. First, culture collections are not random samplings from nature but rather reflect collections of samples that were deposited for archiving because they were considered interesting by someone and were deemed worth saving. Hence, we do not know the extent that such collections represent the diversity in nature but it is likely that they reflect significant biases in what is preserved. Second, the data analyzed was gathered over many years, entered into the archives by different individuals, and it is possible that subtle differences in growth conditions and sample handling introduced variables that would not be a factor if all the isolates had been studied at the same time. Despite these limitations culture collections provide a rich trove of archival information that facilitate studies that would otherwise be impossible or very difficult to do.

## Conclusions

Our work provides the first taxonomic map for identifying fungal species with the potential for mammalian virulence based on their capacity to tolerate higher temperatures. The capacity of some fungi to grow at mammalian temperatures is not monophyletic and a significantly higher proportion of ascomycetous yeasts manifest thermal tolerance relative to basidiomycetous yeasts. The molecular mechanisms responsible for differences in thermal tolerance remain poorly understood and this is an understudied area. Genera with a high proportion of thermally tolerant fungal species represent a potential source of new fungal pathogens for mammals. In addition, adaptation to higher temperature growth by species that are currently not associated with human disease as a result of environmental selection in hot environments represents another potential source of new fungal pathogens. Our results also suggest that fungal species are rapidly adapting to climate warming, with a greater change in basidiomycetous yeasts, which are less thermally tolerant than ascomycetous yeasts and thus at greater stress in hotter ambient temperatures. The finding that thermal tolerance is not monophyletic but rather is widespread through distant genera combined with the recent experience of new catastrophic fungal diseases for frogs and bats suggests the need for increased vigilance for health threats from the fungal kingdom.

## Methods

The source of temperature tolerance information used in this study was the culture collection at the CBS (Utrecht) (for table containing raw data see Additional file 5: Table S1). This was the same collection used in our prior study showing that mammalian temperatures are sufficiently high to restrict the growth of most fungal species [8]. This collection yielded 4,289 strains belonging to 138 genera for which temperature growth information was available at 4°C, 12°C, 15°C, 18°C, 21°C, 25°C, 30°C, 35°C, 37°C, 40°C, 42°C and 45°C. This information was obtained when strains were archived by growing on the most suitable medium, which was generally one of the following: glucose-peptone-yeast agar, potato-dextrose agar, or yeast extract-malt extract agar. A strain was considered to manifest positive growth at a given temperature when a colony was visible without magnification. The set analyzed included ascomycetes and basidiomycetes but not zygomycetes, since there are no yeast in the latter. The CBS collection records included information on the source of the isolate (animals, plants, non-living environmental sources, soils, and so on) as well as miscellaneous physiological data, such the ability to assimilate or grow on sugars, nitrogen and compounds such as 50% D-glucose, 60% D-glucose, methanol, ethanol, D-galactose, L-sorbose, D-glucosamine, D-ribose, D-xylose, L-arabinose, D-arabinose, L-rhamnose, sucrose, maltose, α-α-trehalose, methyl-α-D-glucoside, cellobiose, salicin, arbutin, melibiose, lactose, raffinose, melezitose, inulin, starch, glycerol, erythritol, ribitol, xylitol, L-arabinitol, D-Glucitol, D-Mannitol, Galactitol, myo-Inositol, D-glucono-lactone, 2-keto-D-gluconate, 5-keto-D-gluconate, D-gluconate, D-glucuronate, D-galacturonate, DL-lactate, succinate, citrate, propane 1,2-diol, butane 2,3-diol, quinic acid, D-glucarate, palatinose, D-galactonate, levulinate, L-malic acid, L-tartaric acid, D-tartaric acid, meso-tartaric acid, galactaric acid, uric acid, gentobiose, ethylene glycol, Tween 40, Tween 60, Tween 80, nitrate, nitrite, ethylamine, L-lysine, cadaverine, creatine, creatinine, glucosamine, imidazole, D-tryptophan, D-proline and putrescine. Growth without vitamins, myo-inositol, pantothenate, biotin, thiamin, biotin and thiamin, pyridoxine, pyridoxine and thiamin, and without were also tested as well as growth in the presence of Niacin, para-amino benzoic acid, cycloheximide 0.01%, cycloheximide 0.1%, 10% sodium chloride, 16% sodium chloride, growth at pH 9.5 and growth at pH 3. Other tests included the ability to form starch, acid acetic production, urea hydrolysis, Diazonium Blue B reaction, and the ability to ferment sugars (D-glucose, maltose, methyl-a-D-glucoside, sucrose, α-α-trehalose, melibiose, lactose, cellobiose, melezitose, raffinose, inulin, starch, D-xylose). Antifungal drug *in vitro* susceptibility of 1,698 of the 4,289 strains was determined by a microdilution technique following the procedure proposed by the Antifungal Susceptibility Testing Subcommittee of the European Committee on Antimicrobial Susceptibility Testing [31], with modifications of the temperatures and duration of incubation when necessary. Pure Active powder of known potency of flucytosine (Sigma, Saint Quentin Fallavier, France), fluconazole (Pfizer Inc., Groton, CT, USA), itraconazole (Janssen-Cilag, Issy-les-Moulineaux, France), voriconazole (Pfizer), posaconazole (Merck and Co., Rahway, NJ, USA), terbinafin (Novartis Pharma AF, Basel, Switzerland), caspofungin (Merck and Co.) and amphotericin B (Sigma Chemicals) were used [8].

For each of the strains analyzed in this study, both the ITS (ITS1-5.8S-ITS2) and 26S (D1-D2) loci had been sequenced, which allowed us to classify them according to their genetic background by generating hierarchical UPGMA trees based on pairwise sequence alignments (BioloMICS software, BioAware, Hannut, Belgium). This avoided having to use their genus and species names, since these might be misleading in some particular cases, the large and polyphyletic genus *Candida* being the best example. Correlation analyses were carried out using Analyse-it for Excel [47], using the free statistical environment R [48] or using the statistical functions available in the base and correlogram libraries [49]. The growth behaviors at different temperatures and in different carbon sources were coded as discontinuous numbers ranging from 0 to 2, proportionally to the recorded values, coded as ‘-’, ‘w’ and ‘+’ in the CBS database.

## Additional files

Additional file 1: Figure S1. UPGMA tree of ascomycetous yeasts belonging to the Saccharomycetaceae family obtained from a distance matrix based on pairwise alignments of the ITS (ITS1-5.8S-ITS2) and 26S (D1-D2) loci. The scale bar represents the distance (0.2 means 20% distance) between the nodes of the tree. Additional file 2: Figure S2. UPGMA tree of other unclassified ascomycetous yeasts obtained from a distance matrix based on pairwise alignments of the ITS (ITS1-5.8S-ITS2) and 26S (D1-D2) loci. The scale bar represents the distance (0.2 means 20% distance) between the nodes of the tree. Additional file 3: Figure S3. Diagram depicting the correlation of growth behavior at different temperatures by the fungal species studied. Blue and red colors denote areas of positive and negative correlation, respectively. More intense colors indicate values close to -1.0 (red) or to 1.0 (blue), according to the color scale under the correlogram. This figure corresponds to the analyses depicted in Figure 3 in the text, with a different layout to appreciate the statistical significance. In the lower triangular matrix, correlations are depicted as red or blue colors to indicate negative and positive correlations, respectively. More intense colors indicate values close to -1.0 (red) or to 1.0 (blue), according to the color scale under the correlogram. The upper triangular matrix reports the actual correlation values (larger character) and the upper-lower confidence interval values (smaller characters). Descending diagonal reports the growth temperature. Additional file 4: Figure S4. Diagram depicting the correlation between the growth intensity of the fungal species studied on glucose at different temperatures and the resistance to antifungal drugs. Blue and red colors show positive and negative correlation, respectively. Correlation absolute values are indicated by color intensity. Additional file 5: Table S1. Raw data used in this study.
