# Supplementary figures and images for: Distribution and impact of yeast thermal tolerance permissive for mammalian infection
Source: BMC Biol. 2015 Feb 26;13:18. doi: 10.1186/s12915-015-0127-3 (PMC4381509; doi:10.1186/s12915-015-0127-3)

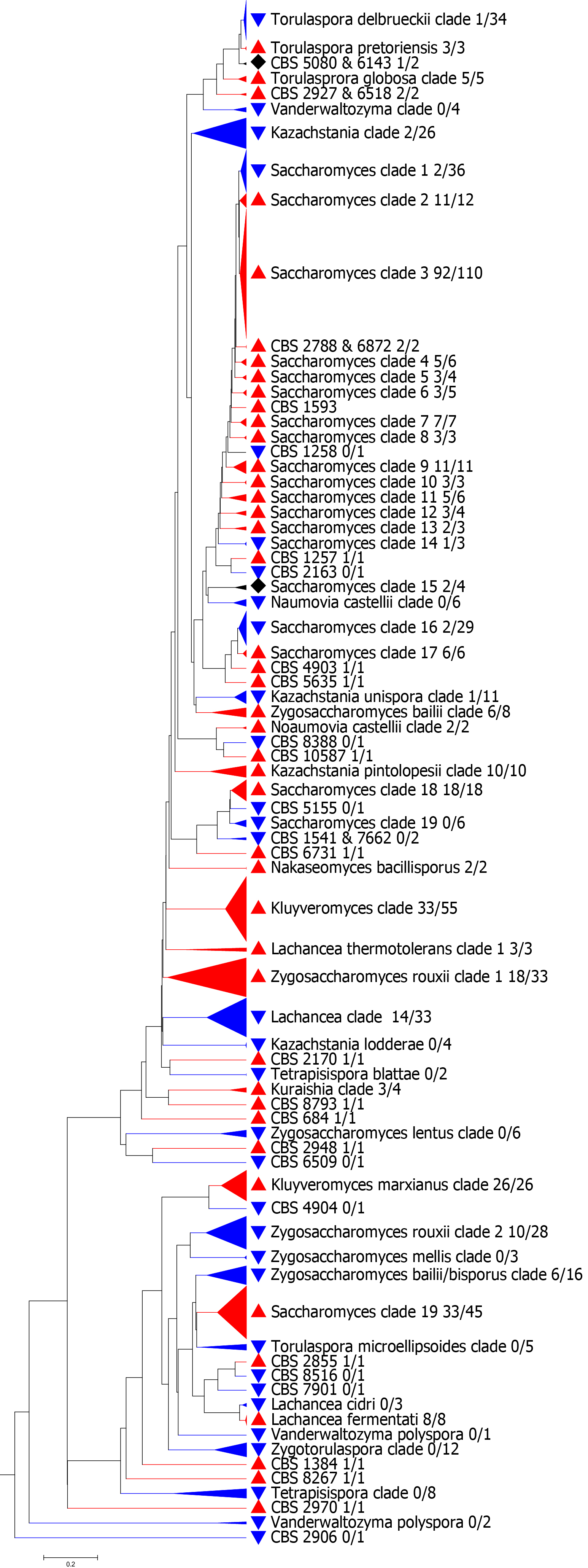

Supplement: Additional file 1: Figure S1. — UPGMA tree of ascomycetous yeasts belonging to the Saccharomycetaceae family obtained from a distance matrix based on pairwise alignments of the ITS (ITS1-5.8S-ITS2) and 26S (D1-D2) loci. The scale bar represents the distance (0.2 means 20% distance) between the nodes of the tree. [file 12915_2015_127_MOESM1_ESM.jpeg]

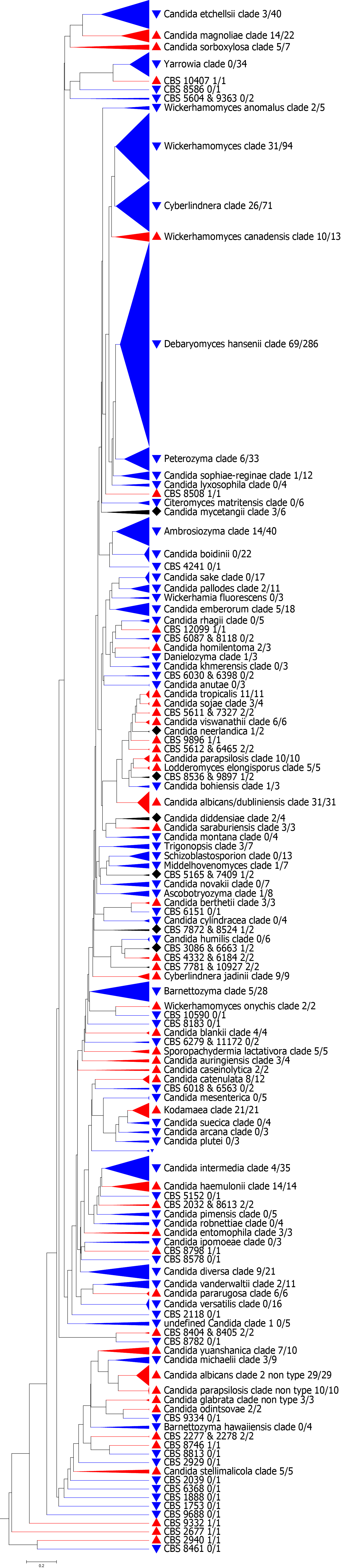

Supplement: Additional file 2: Figure S2. — UPGMA tree of other unclassified ascomycetous yeasts obtained from a distance matrix based on pairwise alignments of the ITS (ITS1-5.8S-ITS2) and 26S (D1-D2) loci. The scale bar represents the distance (0.2 means 20% distance) between the nodes of the tree. [file 12915_2015_127_MOESM2_ESM.jpeg]

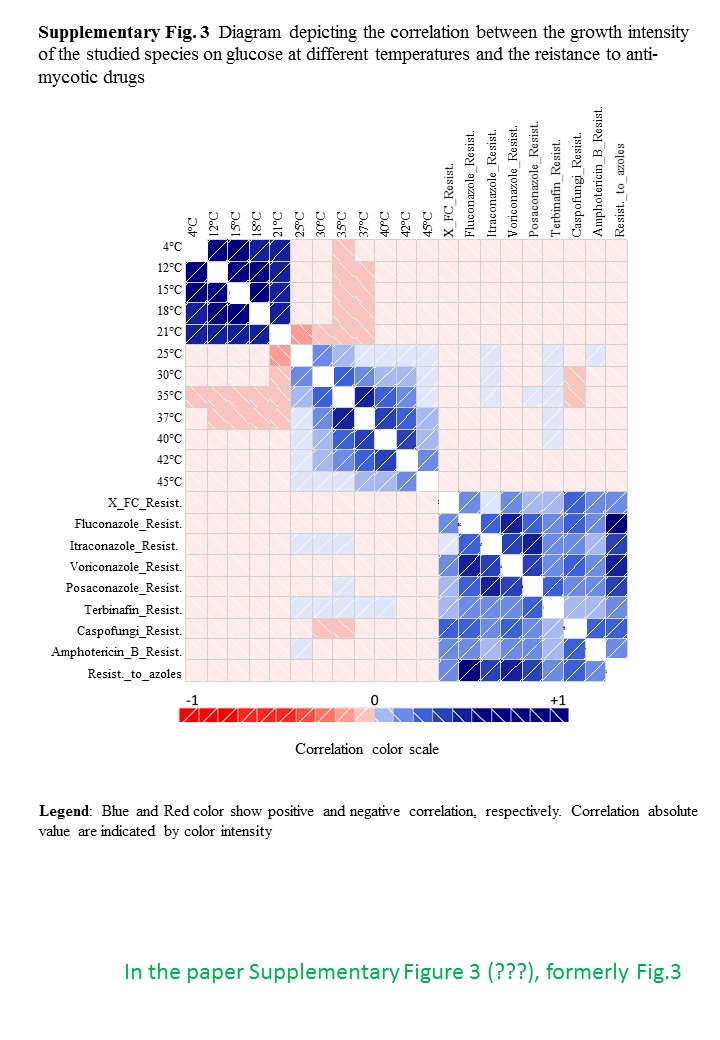

Supplement: Additional file 3: Figure S3. — Diagram depicting the correlation of growth behavior at different temperatures by the fungal species studied. Blue and red colors denote areas of positive and negative correlation, respectively. More intense colors indicate values close to -1.0 (red) or to 1.0 (blue), according to the color scale under the correlogram. This figure corresponds to the analyses depicted in Figure 3 in the text, with a different layout to appreciate the statistical significance. In the lower triangular matrix, correlations are depicted as red or blue colors to indicate negative and positive correlations, respectively. More intense colors indicate values close to -1.0 (red) or to 1.0 (blue), according to the color scale under the correlogram. The upper triangular matrix reports the actual correlation values (larger character) and the upper-lower confidence interval values (smaller characters). Descending diagonal reports the growth temperature. [file 12915_2015_127_MOESM3_ESM.jpeg]

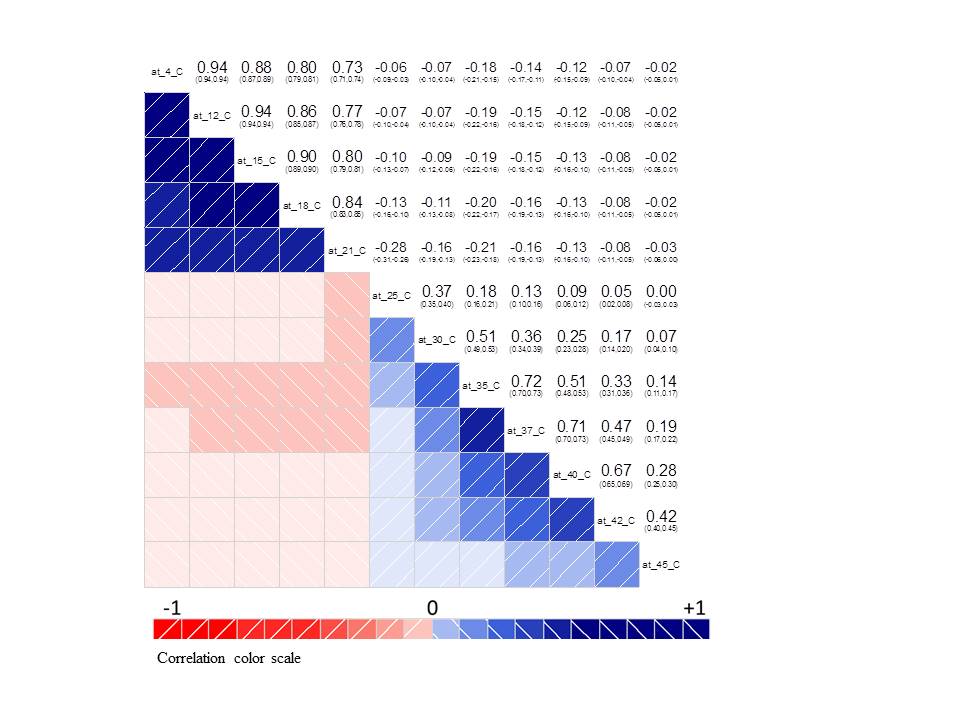

Supplement: Additional file 4: Figure S4. — Diagram depicting the correlation between the growth intensity of the fungal species studied on glucose at different temperatures and the resistance to antifungal drugs. Blue and red colors show positive and negative correlation, respectively. Correlation absolute values are indicated by color intensity. [file 12915_2015_127_MOESM4_ESM.jpeg]
